# EGFR activation in cholangiocytes promotes extrahepatic bile duct regeneration after injury

**DOI:** 10.1097/HC9.0000000000000804

**Published:** 2025-10-14

**Authors:** Ashley N. Calder, Takuki Sakaguchi, Mirabelle Peter, John Tobias, Timothy Frankel, Nataliya Razumilava

**Affiliations:** 1 Department of Internal Medicine, University of Michigan, Ann Arbor, Michigan, USA; 2 Penn Genomics and Sequencing Core, Perelman School of Medicine, University of Pennsylvania, Philadelphia, Pennsylvania, USA; 3 Department of Surgery, University of Michigan, Ann Arbor, Michigan, USA; 4 Rogel Cancer Center, University of Michigan, Ann Arbor, Michigan, USA

**Keywords:** bile duct ligation, cellular proliferation, cholangiopathies, cholestasis, primary sclerosing cholangitis (PSC)

## Abstract

**Background:**

The EGF receptor family of 4 tyrosine kinases, EGFR and ERBB2–4, and their ligands regulate the development and homeostasis of digestive organs including the hepatobiliary system. It promotes intrahepatic cholangiocyte proliferation, bile duct development, and cholangiocarcinoma aggressiveness. The EGF signaling network's contribution to extrahepatic bile duct (EHBD), which is a distinct hepatobiliary entity, regeneration is poorly defined. This work aimed to determine the fundamental role of the EGF signaling network in the biliary proliferative response to EHBD obstruction.

**Methods:**

We used mouse bile duct ligation to model obstructive EHBD injury, and human and mouse EHBD organoids for in vitro studies. We tested activating and inhibitory paradigms with recombinant EGF family ligands and receptor antagonists. Transcriptomic and immunohistochemistry analyses informed EGF signaling changes and cellular localization at homeostasis and after obstruction.

**Results:**

At homeostasis, the EHBD expressed EGFR ligands *Tgfa*, *Btc*, *Hbegf*, and *Nrg4* in cholangiocytes, and *Egf* and *Nrg2* in stromal cells. *Erbb2* and *Erbb3* were predominant receptors expressed in cholangiocytes, and *Egfr* in stromal cells at baseline. Post-injury, biliary hyperproliferation was associated with increased abundance of *Tgfa*, *Btc*, *Hbegf*, and *Areg* ligands and *Egfr* receptor in cholangiocytes with resulting EGFR activation. EGFR ligands induced biliary organoid growth, and inhibition of EGFR, not ERBB2, dampened organoid proliferation. EGFR inhibition in mice led to a decrease in the biliary proliferative response after EHBD obstruction.

**Conclusions:**

The obstruction-induced biliary proliferation is EGFR-mediated, suggesting context-specific and receptor-specific EGF signaling network contribution to EHBD regeneration after injury.

## Graphical abstract

**Figure:**
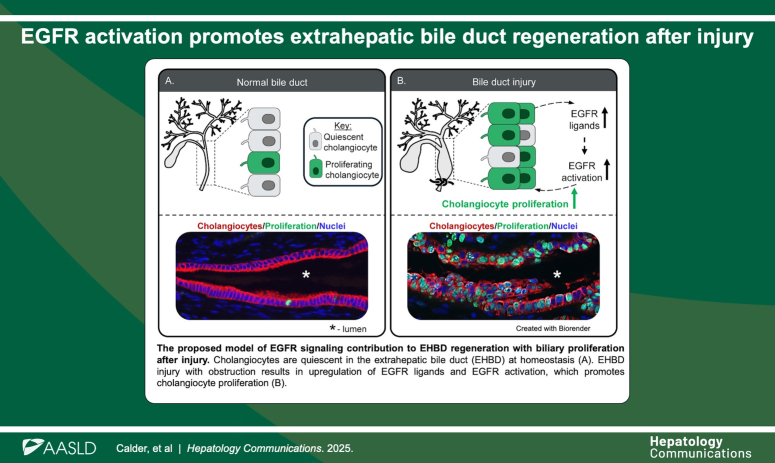

## INTRODUCTION

The biliary tree is the residence of a wide range of pathologies collectively named cholangiopathies. Cholangiopathies are associated with biliary obstruction, inflammation, biliary hyperproliferation, and extracellular matrix remodeling, which can progress to liver fibrosis and failure requiring liver transplant or to biliary cancer.^1^ It is of major importance to develop strategies supporting biliary repair after injury to prevent cholangiopathy development and progression.

Organ regeneration aims to restore the organ function after injury through the recovery of its cellular and structural integrity. Cell proliferation is a common regenerative response to insults. In the hepatobiliary system, liver regeneration is the most studied, in part owing to the liver’s striking ability to recover its pre-resection hepatocyte volume in just 3 weeks.^2^ Cholangiocytes, similar to hepatocytes, are mitotically quiescent at homeostasis, yet they exhibit a prominent proliferative response to obstructive injury, as we and others had shown using a mouse bile duct ligation (BDL) model.^3^^–^^5^ Surprisingly, the mechanisms regulating regeneration and biliary epithelium proliferation in large bile ducts, including the extrahepatic bile duct (EHBD), are less studied. Large perihilar and distal bile ducts compose the EHBD. There are pathological differences in diseases of intrahepatic and extrahepatic bile ducts due to their distinct anatomic location, histological composition, and embryonic origin.^6^ This work aims to uncover fundamental mechanisms supporting EHBD repair through the proliferative response to injury.

Developmental signaling pathways, including WNT, Hedgehog and Hippo signaling, are implicated in large bile duct responses to injury.^7^^,^^8^ Our group has recently reported tightly regulated intercellular communication supporting EHBD proliferation after obstruction.^3^ In this cellular crosstalk, Hedgehog signaling originates from injured cholangiocytes to communicate with receptive fibroblasts, recruiting neutrophils into the damaged bile duct to promote cholangiocyte proliferation. Cholangiocyte autocrine signaling through upregulation of WNT ligands with resulting β-catenin transcriptional programming activation can also support biliary proliferation.^4^ While both Hedgehog and WNT pathways play a role in bile duct regeneration, their inhibition does not completely abrogate the proliferative response of cholangiocytes, suggesting involvement of other mechanisms.

In the past, numerous studies have explored the EGF signaling network in liver biology. EGF signaling consists of 4 receptor tyrosine kinases, EGF receptor (EGFR) and erythroblastoma oncogene B (ERBB) 2–4,^9^ also referred to as human epidermal growth factor receptors (HER1–4) in humans. EGF is a prototypic EGFR signaling ligand. Transforming growth factor-α (TGFα), betacellulin (BTC), heparin-binding EGF-like growth factor (HB-EGF), amphiregulin (AREG), and epiregulin (EREG) ligands have binding specificity for EGFR (TGFα, BTC, HB-EGF, AREG, and EREG) and ERBB4 receptors (BTC, HB-EGF, and EREG).^10^ Notably, ERBB2 lacks a ligand-binding domain on its extracellular region and, therefore, requires heterodimerization with other EGF network receptors. Neuregulin ligands bind ERBB3 (NRG1-2) and ERBB4 (NRG1-4).^10^^–^^12^ In the liver, EGF was shown to promote hepatoblast differentiation into cholangiocytes, bile duct branching, and support biliary organoid cultures.^10^ EGFR and AREG were increased in the livers of patients with primary biliary cholangitis (PBC) and primary sclerosing cholangitis (PSC) associated with biliary hyperproliferation. They protected mice from cholestatic liver injury by supporting cholangiocyte proliferation.^13^ EGFR ablation in partial hepatectomy dampened cellular proliferation and delayed HCC development in mouse models.^14^ Lastly, decreased EGFR turnover in cholangiocytes resulted in sustained EGFR signaling and aberrant biliary hyperproliferation observed in polycystic liver disease and intrahepatic cholangiocarcinoma (CCA).^15^ These studies suggest the role of the EGF signaling network in liver responses to injury and in liver cancer. Despite the body of research on the EGF signaling network in the liver and intrahepatic bile ducts, its status, cellular localization, and effects in EHBD injury are not well described. Accordingly, we hypothesized that the EGF signaling is upregulated in cholangiocytes during obstructive EHBD injury to promote bile duct regeneration with biliary proliferation.

In the present study, we used BDL to model obstructive EHBD injury, as other currently available models of cholestatic liver injury are not known to recapitulate the proliferative phenotype in the EHBD.^16^^,^^17^ We used human and mouse EHBD organoid models to assess the direct effects of EGF family signaling activators and inhibitors on cholangiocytes. We report activation of the EGF signaling network in obstructed EHBDs with increased abundance of EGFR ligands and phosphorylated EGFR (pEGFR) in cholangiocytes. EGFR ligands exhibited a redundant role in the promotion of EHBD organoid proliferation. Through testing of an inhibitory paradigm, we determined dependence of the biliary proliferative response on EGFR, and not ERBB2, activation, suggesting receptor-specific and context-specific effects. Together, our study unveiled the landscape of EGF family signaling components in the EHBDs and showed the prominent role of activated EGFR in promoting EHBD cholangiocyte proliferation in response to biliary obstructive injury.

## METHODS

### Mice

All mouse experiments were designed and conducted in accordance with ARRIVE guidelines and approved by the University of Michigan’s Institutional Animal Care and Use Committee. All mice were bred on a C57BL/6 background and housed in a specific pathogen-free environment with a 12:12-hour light–dark cycle in ventilated caging with Enviro-Dri absorbent or cotton squares as enrichment and provided ad libitum access to food (Purina LabDiet 5L0D) and water. We observed a robust biliary proliferative response in 2–5-month-old mice; thus, all BDL mice were kept within this age range. We used 2–3-month-old mice for EGF inhibition experiments, 3.5–5.5 months old for immunohistochemical staining for KRT19 and phospho-EGFR, and 2–4.8 months old mice for RNA sequencing experiments.^4^ As we did not observe sex-dependent responses to sham/BDL surgeries previously in our bulk RNA-seq analysis,^4^ both male and female mice were used and sex and age matched, when possible, and combined for experimental analyses. Acute obstructive EHBD injury was induced by BDL as was previously described.^3^ In brief, a single ligature was placed at the distal end of the EHBD near the duodenum to mimic specifically EHBD obstruction with dilatation, contrary to the 2, mid-EHBD and proximal EHBD, sutures usually done for liver studies and preventing EHBD dilation.^18^ Animals were euthanized according to institutional guidelines. For pharmacologic inhibition of EGFR signaling, the EGFR inhibitor, erlotinib (LC Laboratories, Cat#: E-4007), was resuspended at 20 mg/mL in PEG400 immediately before the experiment and administered by gavage at 100 mg/kg starting 24 hours before the BDL and 24 hours thereafter. To assess cellular proliferation, 5-ethynyl-2’-deoxyuridine (EdU; Lumiprobe, Cat#: 10540) was prepared according to the manufacturer’s instructions and administered to mice intraperitoneally 2 hours before euthanasia as previously described.^4^

### Human and mouse organoid cultures

EHBD biliary organoid cultures were generated and maintained as previously described with minimal modifications.^4^ In brief, mouse and human EHBDs were minced and dissociated using Liberase TL enzyme blend (Millipore Sigma, Cat#: 5401020001) and grown in organoid growth media containing 10% L-WRN and 30% L-WRN conditioned media for mouse and human organoids, respectively, and 50 μg/mL EGF for human organoid maintenance. Organoids were frozen in media containing 10% DMSO, thawed before use, and passaged at least once before experiments. Organoids between passages 5 and 20 were used for EGF family signaling studies. As we did not observe sex differences in in vivo studies, and cholangiopathies affect both sexes, we used both male and female-derived samples when feasible for all experiments. For experiments with EGF signaling modulators, human and mouse organoids were cultured in EGF-free, serum-free, and conditioned-free medium supplemented with 3 µM CHIR-99021 to maintain WNT signaling (Apex Bio, Cat#: A3011) as indicated in the experimental timelines. Organoids were trypsinized and triturated to disperse organoids into single cells, resuspended at 150 cells/µL concentration in a 75% Matrigel (Corning, Cat#: 354230)/25% DMEM-F12 mixture. Control and treatment wells were seeded from the same organoid cell resuspension. EGFR ligands TGFα (100-16A), BTC (100-50), HB-EGF (100-47), AREG (100-55B), EREG (100-04), and NRG1 (100-03) were purchased from Peprotech and resuspended per the manufacturer’s protocol in 0.1% bovine serum albumin and stored at −20 °C. Ligands or vehicle control (0.1% bovine serum albumin) were diluted on the day of the experiment and added to the culture medium. The initial ligand doses were determined based on the published literature.^19^^,^^20^ For pharmacologic inhibition of EGFR, erlotinib (LC Laboratories, Cat#: E-4007) was resuspended in DMSO at a concentration of 10 mM and stored at −20 °C. Media containing erlotinib or vehicle control (DMSO) were made and added to the culture medium 24 hours after organoid seeding; concentrations were determined based on published literature.^20^ Trastuzumab, an ERBB2 inhibitor, was reconstituted in sterile PBS to a concentration of 10 mg/mL and stored at 4 °C. Medium containing trastuzumab or control (Human IgG isotype) was made and added to culture media 24 hours after organoid seeding; concentrations were chosen based on published literature.^21^ Organoid and cell culture growth was assessed with CellTiter-Glo 3D Viability Assay (Promega, G9681) and the CellTiter-Glo 2.0 (Promega, G924A) Viability Assay, respectively, through measurement of ATP levels as previously described.^4^ Organoid number and growth were assessed by enumeration of all organoids with a visible lumen with ImageJ (NIH) using images from at least 3 wells taken with a stereomicroscope (Olympus, SZX16) and digital microscope camera (Olympus, DP72) using cellSens software (Olympus).

### BT474 breast cancer line cell culture

The immortalized ERBB2-positive breast cancer cell line BT474^21^ was a kind gift of Dr Carole Parent (University of Michigan). Cells were maintained in RPMI culture medium containing 10% fetal bovine serum and 1% Pen-strep and seeded in a 48-well plate at 30,000 cells/well. Media containing 10 μg/mL trastuzumab (MedChemExpress, Cat#: HY-P9907)^21^ or a human IgG isotype (Invitrogen, Cat#: 027102) control was added to cells 24 hours after seeding and replaced every 2 days until the final analysis.

### Immunohistochemistry

Mouse EHBDs were isolated 24 post-BDL, washed in PBS, fixed in 10% neutral buffered formalin (Sigma-Aldrich, Cat#: HT5012) at 4 °C overnight and processed for immunofluorescence analysis as previously described with minor modifications.^22^ Slides were rehydrated and incubated in boiling antigen retrieval buffer for 10 minutes. Antigen unmasking solution (Vector Labs, Cat#: H-3300) was used for all immunofluorescence stains and Trilogy (Sigma-Aldrich, Cat#: 922P-04) was used for KRT19 3,3’-diaminobenzidine (DAB). Primary and secondary antibodies used for immunofluorescence analysis are listed in the Supplemental Table S1, http://links.lww.com/HC9/C111. Hematoxylin and eosin (H&E; Vector Labs, Cat#: H-3502) staining was done following the manufacturer’s protocol (Vector Labs). For DAB staining, Vectastain Elite ABC kit, Peroxidase (Vector Labs, Cat#: PK6100) and DAB substrate kit (Vector Labs, Cat#: SK4100) were used according to the manufacturer’s instructions. Biliary proliferation was assessed by labeling proliferating cells with EdU and using a Click-it Kit (Invitrogen, Cat#: C10337) according to the manufacturer’s protocol. The epithelial cell compartment was determined by positive cytokeratin 19 (KRT19) staining and histological examination. Epithelial and stromal cell compartments were analyzed for the percentage of EdU-incorporating cells as a proportion of DAPI-marked cells in each compartment.

### Transcriptomic analyses

EHBD organoid RNA was extracted as previously described using the Rneasy mini kit (Qiagen, Cat#: 74106) with a DNase treatment (Qiagen, Cat#: 79254) according to the manufacturer’s instructions.^4^ The quantitative reverse-transcriptase polymerase chain reaction (qRT-PCR) analysis of mouse EHBD organoids was performed using iScript cDNA Synthesis kit (Bio-Rad, Cat#: 1708891) for cDNA generation and iTaq Universal SYBR Green Supermix (Bio-Rad, Cat#: 1725121) for mRNA abundance examination. Primer sequences were designed using the NCBI Primer Blast tool (National Library of Medicine) (Supplemental Table S2, http://links.lww.com/HC9/C111) and prepared by Integrated DNA Technologies. Expression data were normalized to 18S ribosomal RNA. All biological samples were analyzed in technical triplicate. For the transcriptomic analysis of the whole mouse EHBD by bulk RNA sequencing (RNA-seq) and single-cell RNA-seq (scRNA-seq), we conducted a secondary analysis of our published datasets GSE280724 and GSE280889.^4^ To evaluate mRNA abundance of EGF family signaling components in human organoids, we mined the published dataset E-MTAB-7569.^23^ Analyses of datasets were performed as previously described.^4^ Dot plots of scRNA-seq datasets were performed in R-studio version 2023.06.1 with the Seurat package version 4.1.1. to visualize gene transcripts within cell populations.

Analysis of published single-cell RNA-seq datasets from liver samples^24^ was performed using the CZ CELLxGENE database and gene expression interpretation tool.^25^ RNA-seq datasets from cholangiocarcinoma and adjacent normal tissue samples from The Cancer Genome Atlas (TCGA) were examined for EGF signaling ligands and receptors.

### Gene Set Enrichment Analysis

Gene Set Enrichment Analysis (GSEA) (v4.3.3) was run in preranked mode with the DESeq2 statistic as the ranking metric for the BDL versus sham contrast in the bulk RNA-seq dataset. Pathways from the hallmark and the c2.cp.biocarta.v2024.1.Hs.symbols collections were scored using 1000 permutations.^26^ Results for pathways of interest were presented individually.

### Statistical analysis

Unpaired *t* test or one-way analysis of variance (ANOVA) was used to determine statistical significance. Values are presented as mean±SD, and *p*-value significance was set at *p*<0.05. Control groups are represented by either vehicle-treated samples or sham procedures. All experiments included at least 3 mice per group for analyses and 3 independent organoid culture lines, except for the discovery of the activating or inhibiting dose of the pharmacologic agent, where at least 3 technical replicates were used. All statistical analyses were performed using Prism v.10 (GraphPad). Significant pathway enrichment was determined using the FDR *q*-value generated from the GSEA software.^26^

## RESULTS

### EGF signaling network is upregulated in obstructed EHBDs

**FIGURE 1:**
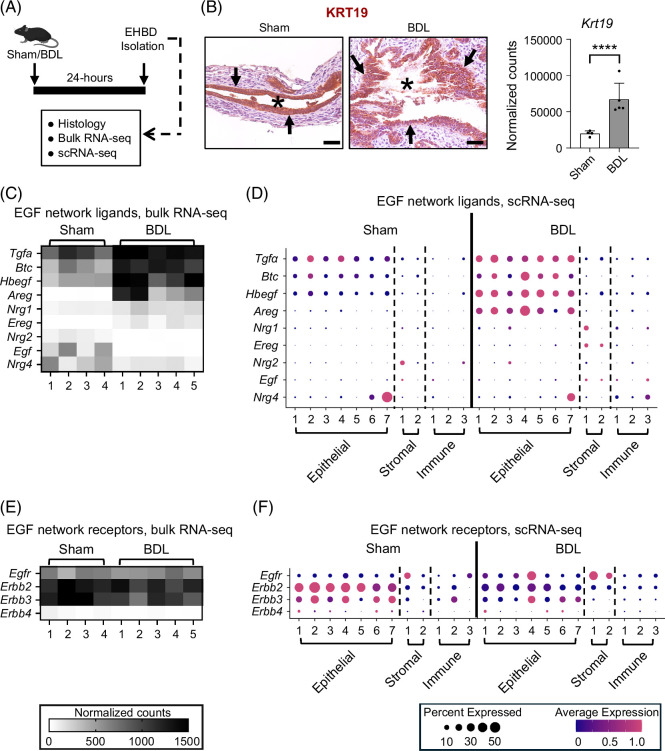
EHBD obstruction results in changes in EGF signaling ligand and receptor expression. Experimental design for WT mice that underwent 24-hour sham or BDL (A). EHBDs were examined for the epithelial marker KRT19 with immunohistochemistry (brown; B, left) and bulk RNA-seq Krt19 gene expression (B, right). EGF signaling ligand (C, D) and receptor (E, F) expression from bulk RNA-seq (left) or scRNA-seq (right) from EHBDs 24 hours after surgery. Asterisks mark EHBD lumen. Histology, n=3 mice/group; bulk RNA-seq, n=4–5 mice/group; scRNA-seq, n=2 samples/treatment, 5 mice/sample. The data are presented as the mean±SD. Statistical significance for bulk RNA-seq was determined using DESeq2. ****p<0.0001. Arrows mark KRT19+ cholangiocytes. Scale bars, 50 μm. Abbreviations: BDL, bile duct ligation; EHBD, extrahepatic bile duct; KRT19, keratin 19; RNA-seq, RNA sequencing; scRNA-seq, single-cell RNA-seq; WT, wild type.

To determine the potential role of the EGF family signaling network in EHBD regeneration, we employed the mouse model of biliary obstruction, BDL, and examined EHBDs at baseline (sham) and post-BDL using histologic and transcriptomic analyses 24 hours after surgery (Figure 1A). We previously reported that the peak mouse EHBD proliferative response occurs at the 24-hour time point.^3^^,^^4^ We analyzed our published scRNA-seq dataset from sham and BDL mouse EHBD cells 24 hours post-surgery,^4^ and confirmed increased expression of proliferation markers (*Mki67*, *Pcna*, *Aurka*, *Ccnd1*, and *Top2a*) in cholangiocyte clusters following BDL (Supplemental Figure S1, http://links.lww.com/HC9/C111). Accordingly, we demonstrated epithelial compartment expansion through an increase in the cholangiocyte marker, KRT19, 24 hours after BDL using immunohistochemistry and bulk RNA-seq analyses (Figure 1B).

To understand the status of the EFG signaling network in homeostatic and injured EHBDs, we analyzed transcriptional expression and cellular localization of ligands with bulk RNA-seq and scRNA-seq. We demonstrated a significant increase in the abundance of *Tgfa*, *Btc*, *Hbegf*, and *Areg* ligand-encoding genes post-BDL with bulk RNA-seq (Figure 1C). scRNA-seq analysis mapped these ligands primarily to epithelial cell clusters at homeostasis and after injury (Figure 1D). While *Nrg1* and *Ereg* were expressed at low levels at homeostasis, mapped to fibroblasts and had a statistically significant increase, their mRNA abundance post-BDL remained low relative to other ligands (Figures 1C, D). Interestingly, *Egf* and *Nrg2* mapped mainly to fibroblasts, and *Nrg4* mapped predominantly to epithelial cells and were expressed at low levels at homeostasis; however, their abundance decreased after injury (Figures 1C, D). Thus, these findings suggested an association between biliary proliferation and an upregulation of EGFR and ERBB4 receptor ligands in injured EHBDs.

Transcriptomic analysis of our bulk and scRNA-seq datasets before and after BDL showed that receptor-encoding genes *Egfr*, *Erbb2*, and *Erbb3* are expressed at high levels in the mouse EHBD at homeostasis (Figure 1E). In cholangiocytes, *Erbb2* and *Erbb3* were the predominant receptors expressed at homeostasis, but their biliary abundance decreased in injured EHBDs. Importantly, *Egfr* abundance increased in cholangiocytes post-BDL, suggesting differences in EGFR localization in the EHBD at homeostasis and after damage (Figure 1F). *Erbb4* expression was almost absent in mouse EHBDs (Figure 1E). These data suggested an upregulation of *Egfr* and its ligands in cholangiocytes in the EHBD injury response.

**FIGURE 2:**
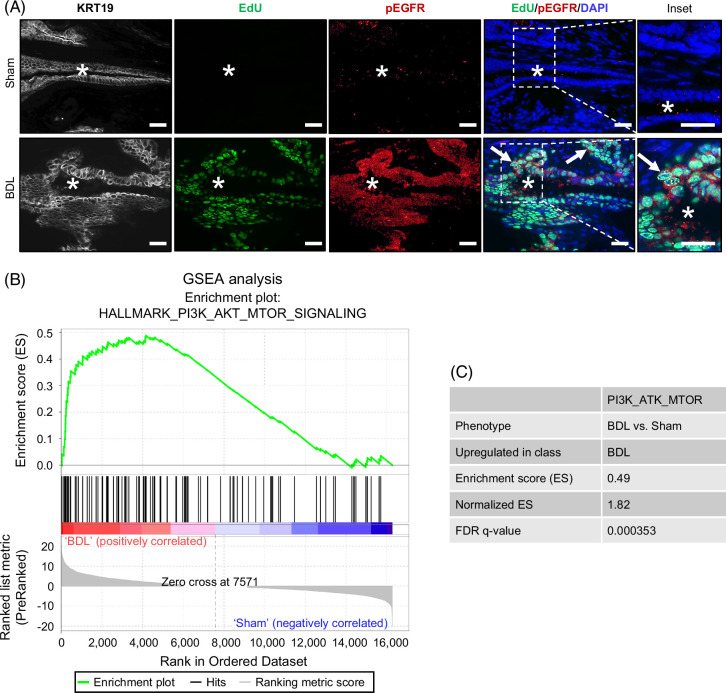
EGFR signaling is activated in the obstructed EHBD. Immunostaining for EGFR activation by phospho-EGFR (Tyr1068) (pEGFR, red), proliferation (EdU, green), and nuclei (DAPI, blue) 24 hours after surgery (A). GSEA using bulk RNA-seq datasets from sham and BDL mouse EHBDs for PI3K/AKT/MTOR signaling (B) and enrichment scores and statistical significance from GSEA analysis (C). Asterisks mark EHBD lumen. Arrows mark pEGFR/EdU co-positive cells. Scale bars, 25 μm. Abbreviations: BDL, bile duct ligation; EdU, 5-ethynyl-2′-deoxyuridine; EGFR, EGF receptor; EHBD, extrahepatic bile duct; GSEA, Gene set enrichment analysis; KRT19, keratin 19; pEGFR, phosphorylated EGFR; RNA-seq, RNA sequencing.

We hypothesized that a surge in ligand expression promotes EGFR activation. As mouse EHBD tissue size prevents effective western blot analysis, we examined sham and BDL mouse EHBDs for phosphorylated (activated) EGFR (pEGFR) by immunostaining. While there was minimal pEGFR at homeostasis, we observed expression of pEGFR with primary localization to cholangiocytes post-BDL (Figure 2A). To further test our hypothesis that EGFR is activated post-BDL, we conducted a gene set enrichment analysis (GSEA) of the bulk RNA-seq datasets from sham/BDL EHBDs with a focus on PI3K/AKT/MTOR and MAP kinase signaling (Figures 2B, C and Supplemental Figure S2, http://links.lww.com/HC9/C111), which are downstream from EGFR.^27^ As anticipated, we observed significant enrichment in PI3K/AKT/MTOR signaling post-BDL as compared with sham EHBDs (Figures 2B, C). There was no statistically significant enrichment in the MAP kinase pathway after BDL (Supplemental Figure S2, http://links.lww.com/HC9/C111). Together, these data supported our hypothesis that injury-induced upregulation of EGFR ligands results in activation of EGFR in cholangiocytes with an increase in PI3K/AKT/MTOR signaling after BDL.

### EGFR-binding ligands promote biliary proliferation in EHBD organoids

To determine the effects of EGFR-binding ligands on EHBD cholangiocytes, we used mouse and human EHBD organoids.^4^^,^^28^ We confirmed that mouse biliary organoids express the genes encoding EGF signaling components, including *EGFR*, using qRT-PCR (Supplemental Figures 3A, B, http://links.lww.com/HC9/C111). To assess the EGF signaling status in human organoids, we analyzed a published bulk RNA-seq dataset^23^ for human biliary organoids (Supplemental Figures 3C–F, http://links.lww.com/HC9/C111). Interestingly, we observed similar expression patterns for EGF signaling ligands, including *TGFA* and *AREG*, and *EGFR*, *ERBB2*, and *ERBB3* receptors, in EHBD and IHBD organoids, suggesting cholangiocytes from different regions of the biliary tree may utilize similar EGF signaling to maintain growth.

**FIGURE 3:**
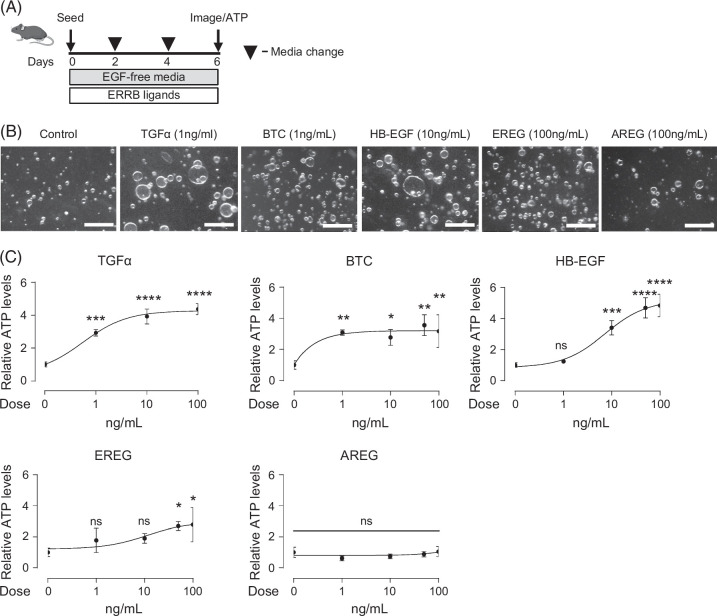
Enhanced growth of mouse EHBD organoids in the dose-dependent response to EGF signaling ligands. Experimental schematic for organoids treated with various EGF signaling ligands (A). Representative bright-field images from control or EGF signaling ligand-treated mouse EHBD organoids (B). Mouse EHBD organoid growth (ATP measurement) was examined in response to a range of doses for EGF signaling ligands TGFα, BTC, HB-EGF, EREG, and AREG (C), n=3–4 technical replicates. A one-way ANOVA with Dunnett multiple comparisons test. The data are presented as the mean±SD. *p<0.05, **p<0.01, ***p<0.001, ****p<0.0001, and ns—not significant. Scale bars, 500 μm. Abbreviations: AREG, amphiregulin; BTC, betacellulin; EGF, epidermal growth factor; EHBD, extrahepatic bile duct; EREG, epiregulin; ERRB, ERBB, erythroblastoma oncogene B; HB-EGF, heparin-binding EGF-like growth factor; TGFα, transforming growth factor-α.

**FIGURE 4:**
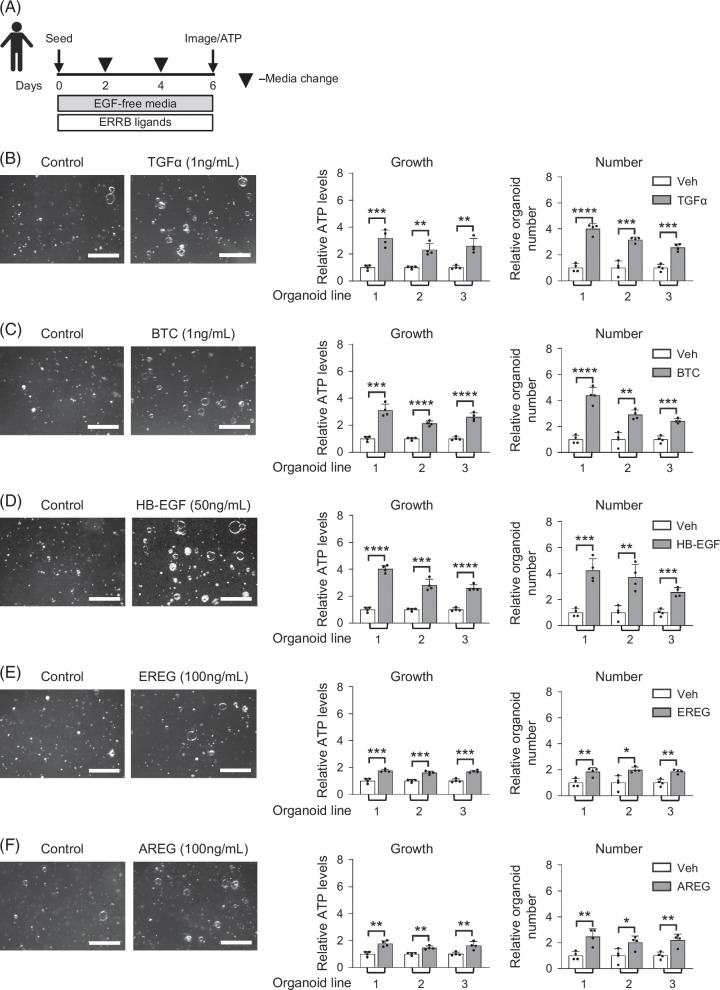
Increased growth and progenitor cell function in human EHBD organoids in response to EGF family signaling ligands. Experimental schematic for organoids treated with various EGF family signaling ligands (A). Human EHBD organoid bright-field images, growth (ATP measurement) and establishment rate (number) were examined in response to recombinant TGFα (1 ng/mL, B), BTC (1 ng/mL, C), HB-EGF (50 ng/mL, D), EREG (100 ng/mL, E), and AREG (100 ng/mL, F) ligands; n=3 technical replicates in 3 organoid lines. Unpaired *t* test compared vehicle to treated for each organoid line. The data are presented as the mean±SD. *p<0.05, **p<0.01, ***p<0.001, ****p<0.0001, and ns—not significant. Scale bars, 500 μm. Abbreviations: AREG, amphiregulin; BTC, betacellulin; EGF, epidermal growth factor; EHBD, extrahepatic bile duct; EREG, epiregulin; HB-EGF, heparin-binding EGF-like growth factor; TGFα, transforming growth factor-α.

We next treated mouse EHBD organoids with recombinant EGFR ligands that were upregulated post-BDL (Figure 3A) and observed a dose-dependent growth (assessed by ATP quantification) response to recombinant TGFα, BTC, HB-EGF, and EREG (Figures 3B, C). These pro-proliferative responses to EGFR ligands were confirmed in 3 different mouse and human biliary organoid lines (Supplemental Figure S4, http://links.lww.com/HC9/C111, and Figure 4). We also observed significant increases in organoid establishment rates (organoid number) from BTC and EREG ligands in mice and all ligands in humans, suggesting that these ligands support progenitor cell function (Supplemental Figures S4C, E, http://links.lww.com/HC9/C111, and Figures 4B–F). Upon treatment with different EGFR ligands, organoids maintained their standard cystic phenotype. Notably, the pro-proliferative effect required 10–100-fold lower concentrations of TGFα, BTC, and HB-EGF (Supplemental Figures S3B–D, http://links.lww.com/HC9/C111) as compared with EREG and AREG (Figures 3E, F), with the latter showing minimal effect even at the higher dose in mouse organoids. This was consistent with prior work showing different affinities of EGFR ligands.^12^ We next tested the effects of NRG1, the ligand for ERBB3, as it was the only neuregulin significantly increased post-BDL, and observed increased organoid growth at high NRG1 concentrations (Supplemental Figure S5, http://links.lww.com/HC9/C111). Thus, our data suggested the interchangeable role of EGF ligands upregulated post-BDL in the promotion of biliary proliferation in organoids.

### Inhibition of EGFR and not ERBB2 abrogates EHBD biliary proliferation in biliary organoids

**FIGURE 5:**
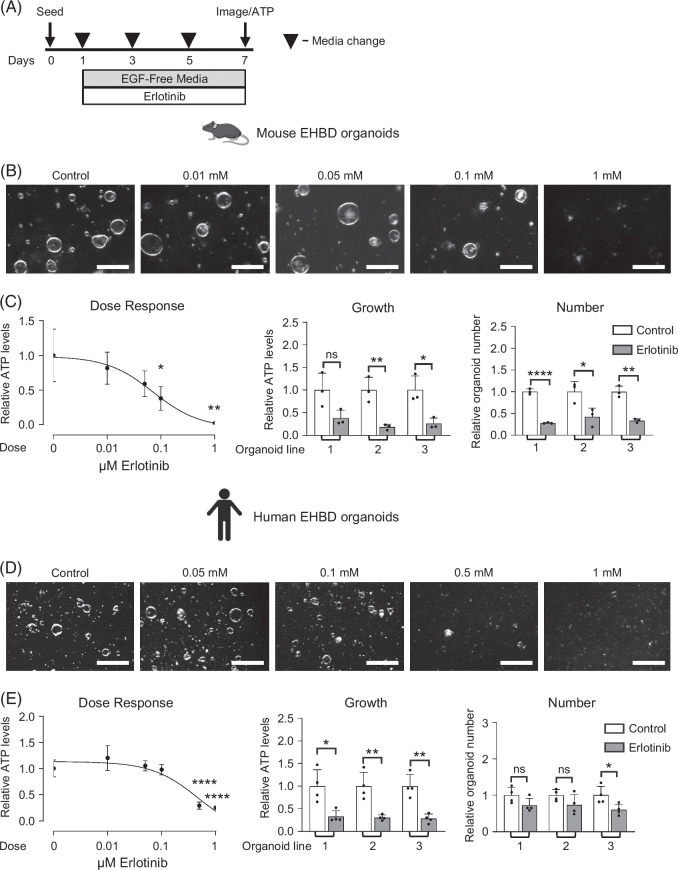
EGFR inhibition attenuates mouse and human EHBD organoid growth. Experimental schematic for organoids treated with the EGFR inhibitor erlotinib (A). Mouse EHBD bright-field images and organoid growth (ATP measurement) were examined in response to a range of erlotinib doses (B and C, left; n=3 technical replicates). Mouse EHBD organoid growth and number in response to erlotinib (0.1 μM), n=3 technical replicates in 3 organoid lines (C, middle and right). Bright-field images and dose–response curve of human EHBD organoids treated with a range of erlotinib concentrations (D and E, left; n=3 technical replicates). Growth and number of human EHBD organoids in response to erlotinib (0.5 μM), n=3 technical replicates in 3 organoid lines (E). One-way ANOVA with Dunnett Multiple Comparisons test (C, E left); and unpaired *t* test compared ligand-treated to vehicle-treated control for each organoid line (E, middle and right). The data are presented as the mean±SD. *p<0.05, **p<0.01, ****p<0.0001, and ns—not significant. Scale bars, 500 μm. Abbreviations: EGFR, epidermal growth factor receptor; EHBD, extrahepatic bile duct.

TGFα, BTC, HB-EGF, AREG, and EREG bind to the EGFR extracellular domain and can transmit downstream signals either through EGFR homodimers or through heterodimerization with other EGF family receptors.^29^ NRG1 binds to ERBB3 and ERBB4. However, ERBB3 lacks kinase activity and relies on heterodimerization with other EGF network receptors for downstream signaling.^29^ As ERBB4 abundance in EHBDs and cholangiocytes was almost undetectable (Figure 1F and Supplemental Figure S3D, http://links.lww.com/HC9/C111), we focused on the contribution of EGFR and ERBB2 to the regulation of biliary proliferation by testing an inhibitory paradigm in organoids. EHBD organoids actively proliferate at baseline, unlike quiescent in vivo cholangiocytes at homeostasis,^4^^,^^22^ and both mouse and human biliary organoids expressed *Tgfa*, *Btc*, *Hbegf*, *Areg*, and *Ereg* ligands, and *Egfr* and *Erbb2* receptors (Supplemental Figure S3A–D, http://links.lww.com/HC9/C111). Thus, we hypothesized that cholangiocytes in organoids maintain their proliferative state through autonomous activation of EGFR and/or ERBB2 signaling. To inhibit EGFR, we chose erlotinib, a small molecule antagonist of EGFR phosphorylation and Food and Drug Administration (FDA) approved for human cancers.^30^ While kinase inhibitors can have off-target effects, erlotinib was shown to have high potency and sensitivity against EGFR over other ERBB receptors and kinases.^31^ Erlotinib decreased growth in mouse and human biliary organoids with an IC^50^ of 0.073 and 0.5 µM, respectively (Figure 5). Erlotinib also decreased organoid numbers in mouse organoids (Figures 5B, C). These findings further confirmed the direct involvement of EGFR signaling in the promotion of cholangiocyte proliferation.

**FIGURE 6:**
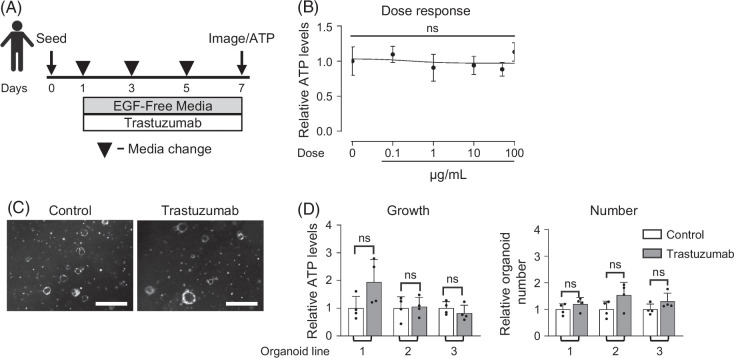
ERBB2 inhibition does not alter human EHBD organoid growth. Experimental schematic for human organoids treated with the ERBB2 inhibitor trastuzumab (A). Dose–response curve for trastuzumab treatment concentrations from 0.1 to 100 μg/mL (n=3–4 technical replicates) (B). Human EHBD organoid bright-field images, growth, and number after treatment with either control (isotype) or trastuzumab (50 μg/mL, C) in 3 biological replicates. One-way ANOVA with Dunnett Multiple Comparisons test (B); unpaired *t* test compared vehicle to treated for each organoid line (D). The data are presented as the mean±SD. ns—not significant. Scale bars, 500 μm. Abbreviation: EHBD, extrahepatic bile duct.

ERBB2 lacks an extracellular ligand-binding domain, however it can be activated by EGFR ligands through dimerization with EGFR.^29^ Therefore, to evaluate the potential contribution of ERBB2 to biliary proliferation, we used trastuzumab, an FDA-approved recombinant humanized anti-ERBB2 antibody^32^ (Figure 6A). We utilized human organoids for this experiment, as trastuzumab does not target mouse cells. Trastuzumab had no effect on human biliary organoid growth or cell number (Figures 6B–D), while it effectively inhibited the growth of ERBB2-overexpressing breast cancer cells,^21^ which were used as a positive control (Supplemental Figure S6, http://links.lww.com/HC9/C111). This suggested that ERBB2 is not required for biliary organoid proliferation.

### Inhibition of EGFR activation in mice results in the dampened biliary proliferative response in obstructed EHBDs

**FIGURE 7:**
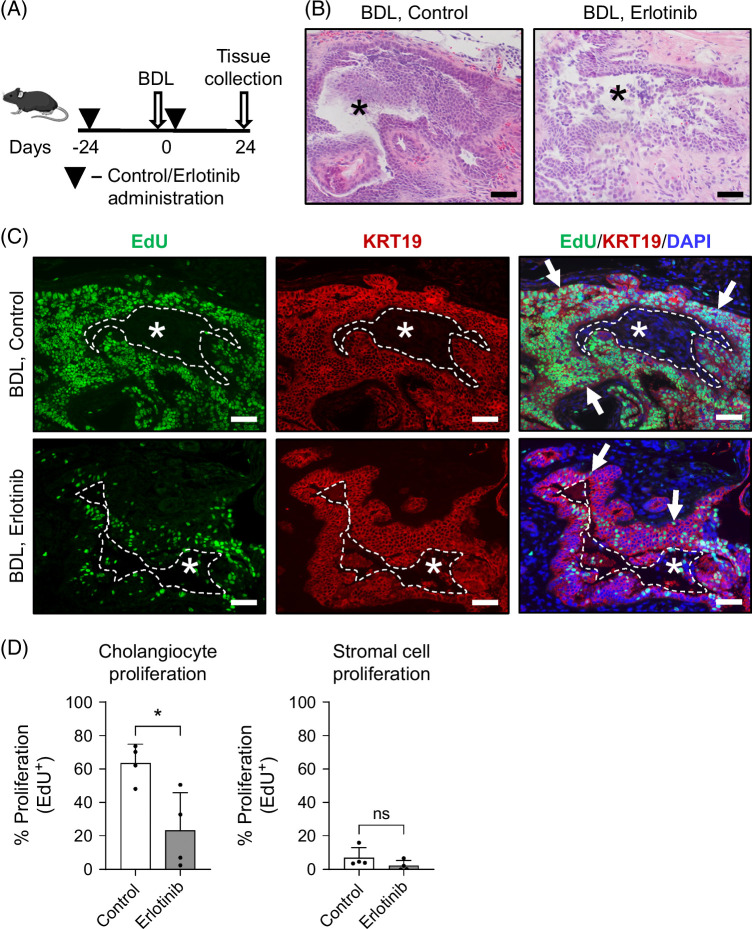
EGFR inhibition decreases cholangiocyte proliferation in injured mouse EHBDs. Experimental schematic for mice treated with vehicle (control) or EGFR inhibitor erlotinib and undergoing BDL surgery (A). H&E images of mouse EHBDs 24 hours post-BDL surgery (B). Mouse BDL sections examined with immunofluorescence for proliferation (EdU, green), KRT19 (cholangiocytes, red), and nuclei (DAPI, blue) (C). Morphometric analysis of proliferation in the epithelial (KRT19+ cholangiocyte) and stromal compartments (D), n=4 mice/group; unpaired *t* test. The data are presented as the mean±SD. *p<0.05 and ns—not significant. Asterisks mark EHBD lumen. Arrows mark proliferating cholangiocytes. Scale bars, 50 μm. Abbreviations: BDL, bile duct ligation; EdU, 5-ethynyl-2′-deoxyuridine; EHBDs, extrahepatic bile ducts; EGFR, epidermal growth factor receptor; H&E, hematoxylin and eosin; KRT19, keratin 19.

We demonstrated upregulation of EGFR signaling in ligated mouse EHBDs in association with induction of biliary proliferation (Figures 1, 2). We also showed that EGFR signaling promotes cholangiocyte proliferation in vitro (Figures 3–5). To determine if EGFR activation is required for the biliary proliferative response in vivo, we treated mice undergoing BDL with the FDA-approved EGFR inhibitor erlotinib^30^ (Figures 7A, B). Erlotinib-treated mice that underwent BDL exhibited a significant decrease in the number of proliferating (EdU+) cholangiocytes (KRT19+) as compared with vehicle-treated BDL mice (Figures 7C, D). Thus, we demonstrated that EGFR signaling promotes EHBD regeneration through support of biliary proliferation after BDL-induced EHBD injury.

To confirm if our findings are relevant to human cholangiopathies, which are known to be associated with biliary hyperproliferation,^33^^,^^34^ we mined published datasets from human normal, PBC, PSC, and CCA hepatobiliary samples. Notably, PBC affects mainly small IHBDs, while PSC mostly affects large IHBD and EHBD bile ducts.^35^^,^^36^ Our analysis of scRNA-seq datasets from PBC and PSC livers^24^^,^^25^ revealed increased expression of *TGFA*, *BTC*, *AREG*, and *NRG1-4* ligands, and *EGFR* receptor in cholangiocytes. The TCGA database analysis showed increased abundance of *TGFA*, *BTC*, and *ERBB3*, while *EGFR* was minimally decreased in CCA as compared with normal tissues (Supplemental Figure S7, http://links.lww.com/HC9/C111). These data suggest EGF family signaling members are upregulated in human cholangiopathies and biliary cancer.

## DISCUSSION

This study investigated the role of EGF family signaling in the cholangiocyte proliferative response to obstructive EHBD injury. While there is a body of work focused on the regulation of epithelial cell responses to injury in the liver and intrahepatic bile ducts, mechanisms regulating EHBD responses to damage are poorly described. Understanding the regulation of cholangiocyte proliferation can inform approaches to promote biliary regeneration and unveil potential mechanisms of uncontrolled proliferation during biliary carcinogenesis. Our study implicated EGFR signaling in EHBD regeneration after bile duct obstruction by contributing to the biliary proliferative response. This effect involves upregulation of EGFR ligand abundance and phosphorylation of the EGFR receptor in cholangiocytes with enrichment of the downstream signaling pathways, especially along the PI3K/AKT/MTOR axis.

Several signaling pathways, including WNT, Hedgehog, and now EGF, have been shown to contribute to EHBD injury-induced proliferation.^3^^,^^4^ The inhibition of any one of these pathways does not completely abrogate injury-induced biliary proliferation, supporting the importance of this response. Moreover, crosstalk between WNT and EGFR signaling in the liver has been suggested based on increased EGFR expression and activation when β-catenin (a WNT effector) is overexpressed.^37^ Notably, in our biliary organoids, which are proliferating cholangiocytes cultured in the presence of WNT,^4^ EGFR expression was comparable to ERBB2 and ERBB3. Thus, organoids more closely resemble post-BDL regenerative cholangiocytes when WNT signaling is elevated and cholangiocytes are proliferating rather than homeostatic quiescent cholangiocytes.^4^ Future studies exploring the crosstalk between these pathways in the orchestration of the cholangiocyte proliferative response are warranted and may provide additional insight into potential targets for biliary diseases. Additional investigations would also need to be focused on the upstream events leading to activation of these signaling pathways, such as changes in bile acid exposure and composition,^38^ as well as effects of mechanical pressure occurring during biliary obstruction.

The activation of EGFR by ligands can elicit distinct ligand, organ system, and pathologic condition-dependent responses.^39^ In the gut, EREG was shown to promote an intestinal organoid phenotype with striking villus-like structures, while exposure to EGF was shown to stimulate formation of progenitor-like cystic organoids.^19^ However, in EHBD organoids, we observed a cystic structure independent of which EGFR ligand was used, suggesting tissue-specific roles for EGF signaling in epithelial cells. Beyond cell proliferation, EGF signaling can regulate other injury responses. Thus, in the liver, EGF and EGFR inhibition had antifibrotic effects in long-term BDL-induced injury. Long-term BDL-induced AREG and HB-EGF expression were shown to protect against hepatocellular injury and fibrosis, respectively.^13^^,^^33^^,^^40^^,^^41^ These studies, coupled with our findings, suggest EGF family signaling has several roles and is important for both the acute and chronic injury responses to biliary obstruction in the liver and EHBD. While most studies in the liver mainly focused on hepatocyte analyses, our work showing a similar pattern of EGF signaling component expression in EHBD-derived and IHBD-derived organoids suggests that IHBD cholangiocytes might respond to biliary obstruction with EGF signaling-induced proliferation in a similar manner.

In the EHBD, we showed that all EGFR ligands that were overexpressed in obstructed EHBDs promoted cholangiocyte proliferation in human and mouse EHBD organoids. We also observed that traditional high-affinity ligands^42^ EGF, TGFα, BTC, and HB-EGF induced cholangiocyte proliferation at lower concentrations than low-affinity ligands, EREG and AREG. However, BTC and EREG, but not TGFα and HB-EGF, also increased organoid establishment efficiency in mouse organoids, suggesting potential BTC and EREG roles in mouse biliary progenitor cell maintenance. Human organoids mounted more prominent responses to the EGFR ligand, AREG, as compared with mouse organoids in our studies. This may suggest that both species-specific differences and the culture environment could affect sensitivity to EGF signaling ligands, especially as human organoids are traditionally cultured in the presence of EGF.^23^ Interestingly, although *Areg* expression increased substantially post-BDL, recombinant AREG had limited effects on mouse organoid growth, indicating that it might regulate other responses to cholangiocytes beyond proliferation. Surprisingly, the abundance of *Egf*, the prototypic EGFR ligand implicated in liver diseases and often used for organoid media, was relatively low and mapped to EHBD fibroblasts and immune cells at homeostasis, and *Egf* abundance significantly decreased in obstructed EHBDs. This suggested that EGF may not be important for responses to acute EHBD injury but instead may play a more important role during homeostasis.^43^ This study provides the basis for further exploration into the roles of specific EGFR ligands in cholangiocyte progenitor cell function and differentiation during homeostasis and in other pathological conditions, including more chronic injury.

EGF family signaling is implicated in human cholangiopathy pathogenesis.^33^^,^^44^^–^^46^ In biliary atresia, elevated EGF levels correspond with the degree of liver fibrosis, and the EGFR pathway is enriched in immune cells populating fibrotic regions of the liver.^33^^,^^44^ Our secondary analysis of cholangiocyte populations in livers of PSC and PBC patients showed increased expression of *TGFA*, *AREG*, and *EGFR* in cholangiocytes, similar to our findings in the obstructed mouse EHBDs. Upregulation of EGFR signaling in cholangiocytes has been also reported in livers of patients with PSC.^45^ EGF signaling upregulation is strongly associated with CCA, where studies reported *EGFR* overexpression in 8.1% of biliary tract cancers, *ERBB2* amplification in 17.2% of perihilar CCA and overexpression of both *EGFR* and *ERBB2* in 7% of biliary cancers. *ERBB2* amplifications were reported to be more common in extrahepatic CCA as compared with intrahepatic CCA (17.4% and 4.8%, respectively), further highlighting the difference in intrahepatic and extrahepatic bile duct biology.^47^ Similar to PBC, PSC, and injured mouse EHBDs, *TGFA* ligand was upregulated in TCGA dataset CCAs, where *ERBB3* abundance was also increased. This observation highlights that EGF ligand upregulation might also contribute to CCA biology. Together, these findings suggest that cholangiocytes are a direct target of EGF signaling in biliary disorders and that EGF signaling might contribute to biliary hyperproliferation observed in cholangiopathies and biliary cancer.

The current study showed that inhibition of EGFR with erlotinib decreased the cholangiocyte proliferative response to EHBD injury. Erlotinib also inhibited the growth of human and mouse biliary organoids, further suggesting that EGFR activation is important for cholangiocyte proliferation. We considered that ligands activating EGFR could potentially signal through EGFR/ERBB2 heterodimers, as ERBB2 lacks a ligand-binding extracellular subdomain and needs to pair with EGFR and ERBB3 for ligand-mediated signals.^48^ While ERBB3 depends on binding with other EGF family receptors for kinase activity, it could potentially bind with ERBB2. As treatment with ERBB2 inhibitor trastuzumab had no effect on biliary organoid proliferation, we concluded that it is likely EGFR, and not ERBB2 or ERBB3, that conducts pro-proliferative signals in EHBD cholangiocytes. Interestingly, a recent phase III clinical trial suggested survival benefits from the addition of erlotinib to a combination of gemcitabine and oxaliplatin for patients with CCA.^49^ In contrast, the inhibition of ERBB2 in CCA yielded disappointing results.^50^ In summary, our comprehensive set of experiments using transcriptomic and functional analyses of a mouse model of obstructive bile duct injury, along with human and mouse organoid models, demonstrated an essential need for EGFR activation in the promotion of the biliary proliferative response in injured EHBDs. We also demonstrated that many EGFR ligands are upregulated to support this regenerative response. By defining the status and landscape of EGF family signaling in the healthy and diseased EHBD, we provide the foundational framework for further studies focused on the EHBD.

## Supplementary Material
