# Supplementary material for: EGFR activation in cholangiocytes promotes extrahepatic bile duct regeneration after injury
Source: Hepatol Commun. 2025 Oct 14;9(11):e0804. doi: 10.1097/HC9.0000000000000804 (PMC12520223; doi:10.1097/HC9.0000000000000804)
Supplement: Supplementary file 1 [file hc9-9-e0804-s001.pdf]

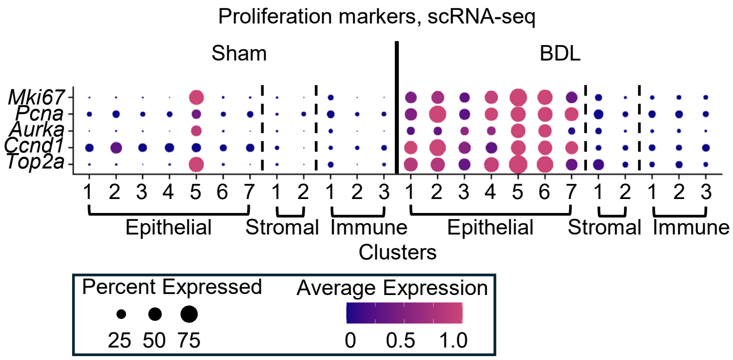

Suppl. Fig. 1: Increase in genes encoding proliferation markers in cholangiocytes after BDL. Analysis of proliferation markers in scRNA-seq in sham and BDL mice 24-hours post surgery. sc-RNA-seq, n = 2 samples/treatment, 5 mice/sample.

A.

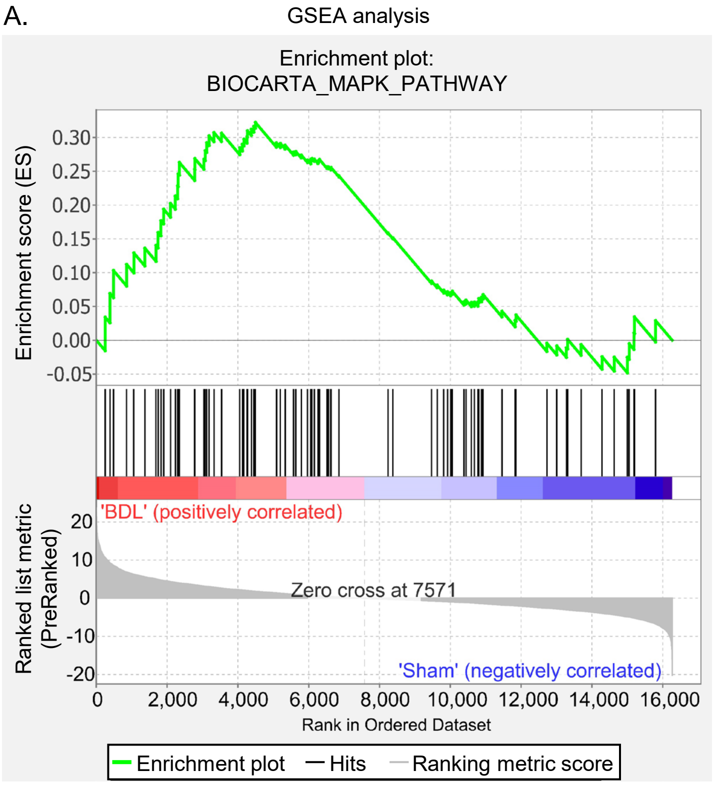

B.

	MAPK
Phenotype	BDL vs. Sham
Upregulated in class	BDL
Enrichment score (ES)	0.32
Normalized ES	1.18
FDR q-value	0.545

Suppl. Fig. 2: MAPK pathway is not significantly enriched following BDL. Gene set enrichment analysis (GSEA) using bulk RNA-seq datasets from sham and bile duct ligated (BDL mouse extrahepatic bile ducts (EHBDs) for the MAPK pathway (A) and enrichment scores and statistical significance from GSEA analysis (B).

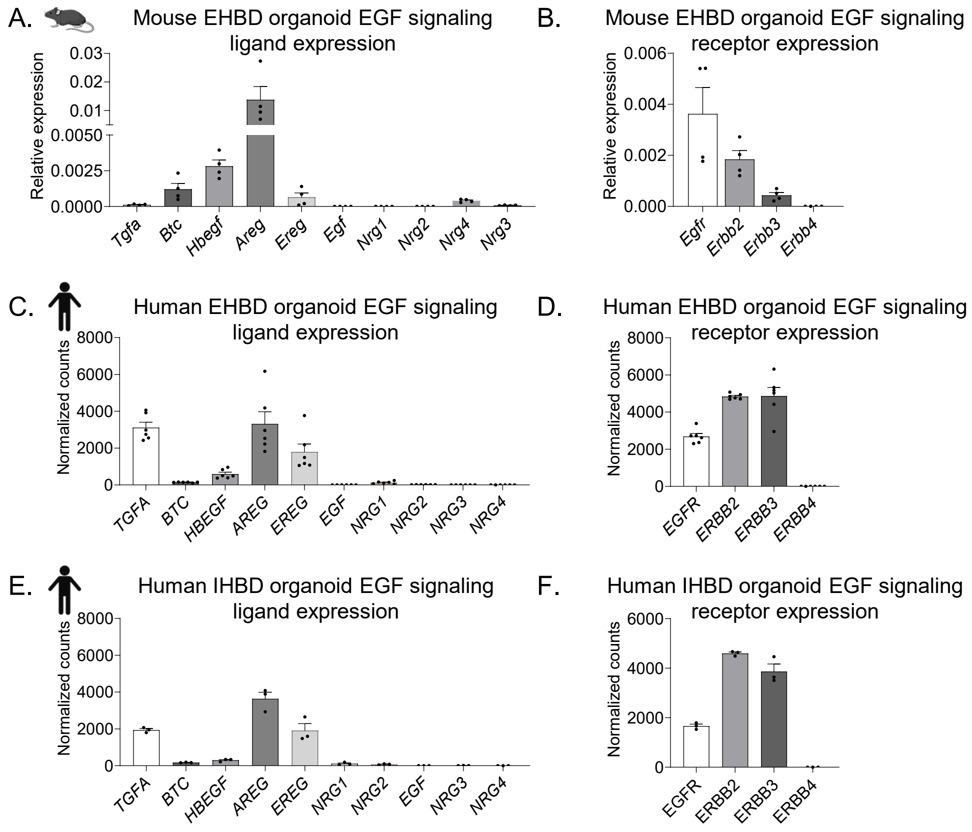

Suppl. Fig. 3: Genes encoding EGF signaling receptors and ligands are expressed in mouse and human EHBD organoids. qRT-PCR for epidermal growth factor (EGF) signaling ligands (A) and receptors (B) in mouse extrahepatic bile duct (EHBD) organoids. Analysis of public dataset (E-MTAB-7569) for EGF family ligands and receptors in human EHBD (C-D) and IHBD (E-F) organoids. Normalized counts from human organoid data was determined using DESeq2.

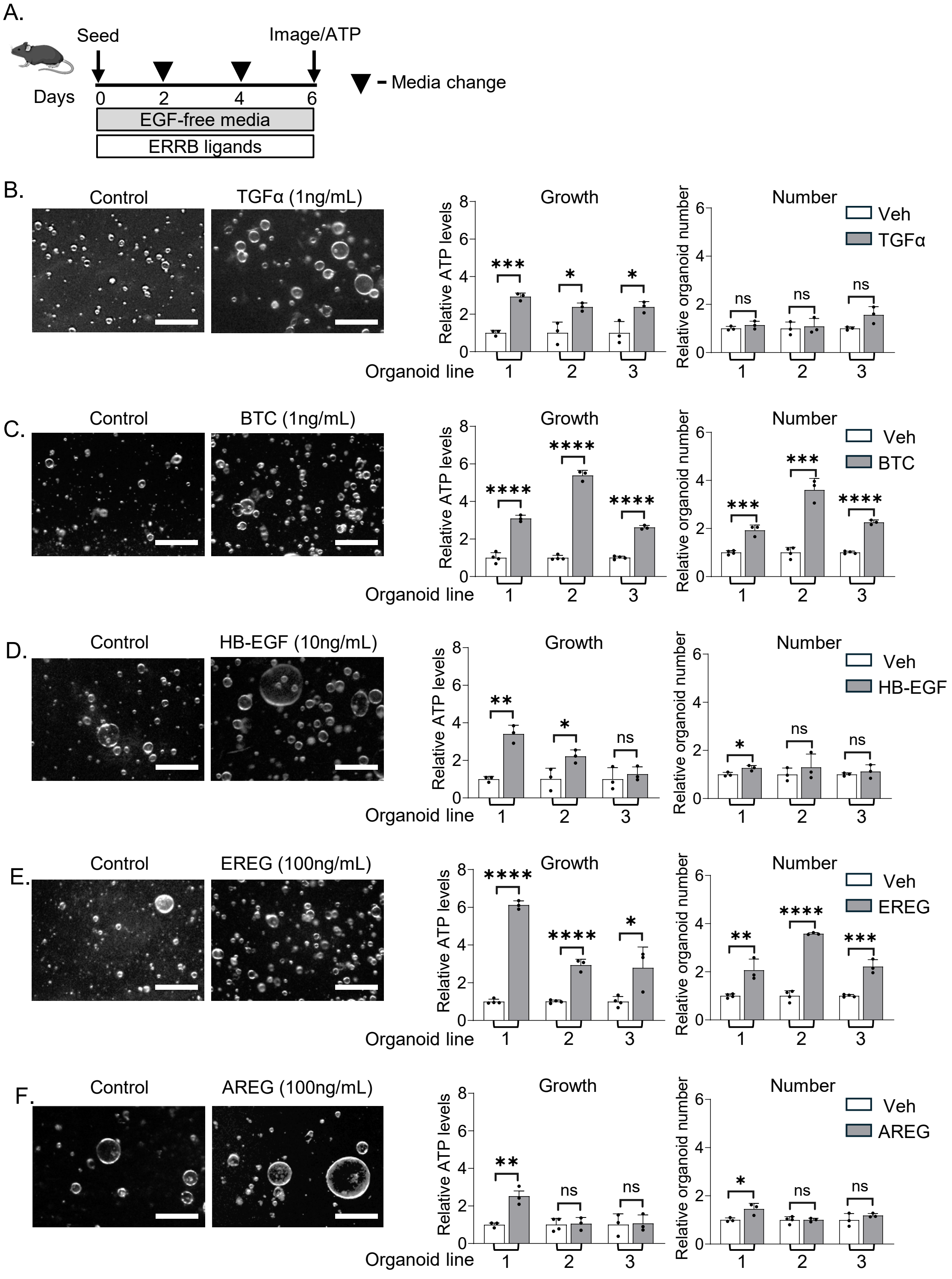

Suppl. Fig. 4. EGF signaling ligands induce growth and progenitor cell function in mouse EHBD-derived organoids. Experimental schematic for organoids treated with various epidermal growth factor (EGF) family signaling ligands (A). Mouse extrahepatic bile duct (EHBD) organoid bright-field images, growth (ATP measurement) and establishment rate (number) were examined in response to recombinant TGFα (1ng/mL, B), BTC (1ng/mL, C), HB-EGF (10ng/mL, D), EREG (100ng/mL, E), and AREG (100ng/mL, F) ligands. n = 3 biological replicates. Unpaired *t*-test compared ligand-treated to vehicle-treated control for each organoid line. The data are presented as the mean ± SD. *$P < 0.05$, **$P < 0.01$, ***$P < 0.001$, ****$P < 0.0001$, ns – not significant. Scale bars, 500μm.

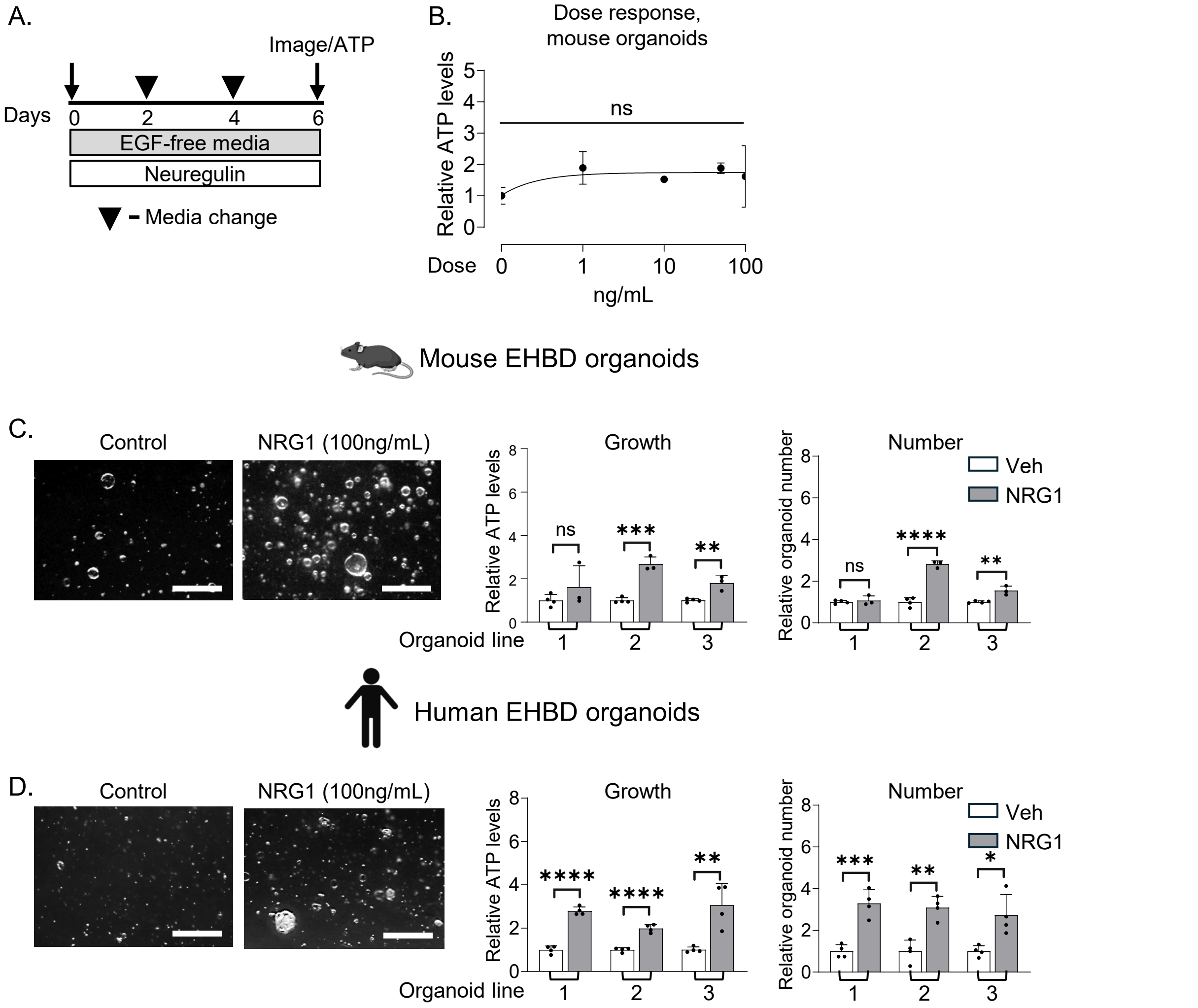

Suppl. Fig. 5. Increased growth of mouse and human EHBD organoids with the ERBB3 ligand NRG1. Experimental schematic for organoids treated with the ERBB3 ligand, NRG1 (A). Dose response for NRG1 in mouse extrahepatic bile duct (EHBD) organoids (B). Bright-field images, organoid growth, and establishment rate (number) were examined in mouse (C) and human (D) EHBD organoids treated with recombinant NRG1 (100ng/mL). n = 3-4 technical (B, dose-response curve in mouse organoids) and n = 3-4 biological (C and D) replicates. One-way ANOVA with Dunnett's Multiple Comparisons test (B); and unpaired *t*-test compared ligand-treated to vehicle-treated control for each organoid line (C,D). The data are presented as the mean ± SD. **P* < 0.05, ***P* < 0.01, ****P* < 0.001, *****P* < 0.0001, ns – not significant. Scale bars, 500μm.

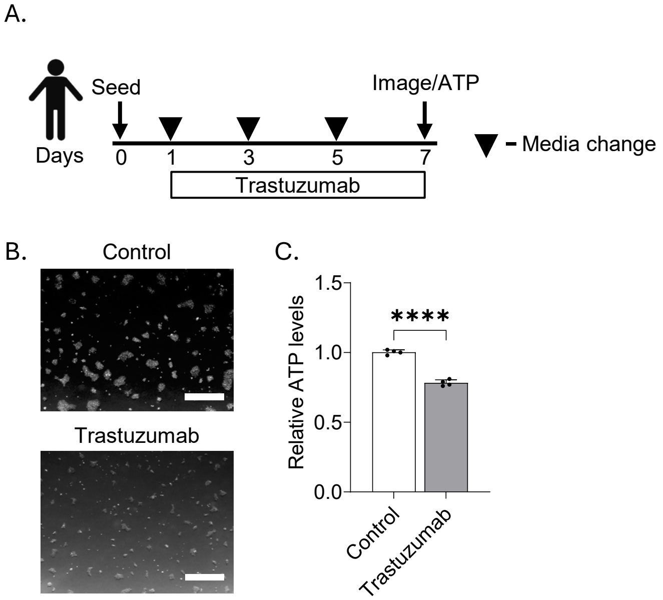

Suppl. Fig. 6. Trastuzumab inhibits growth in ERBB2 overexpressing breast cancer cells. Experimental schematic for treatment of ERBB2 overexpressing breast cancer cell line, BT474, with ERBB2 inhibitor trastuzumab. Bright-field images (B) and growth (C) in cells treated with control (isotype) or trastuzumab (10ug/mL). Unpaired *t*-test. The data are presented as the mean ± SD. ns – not significant. Scale bars, 500μm.

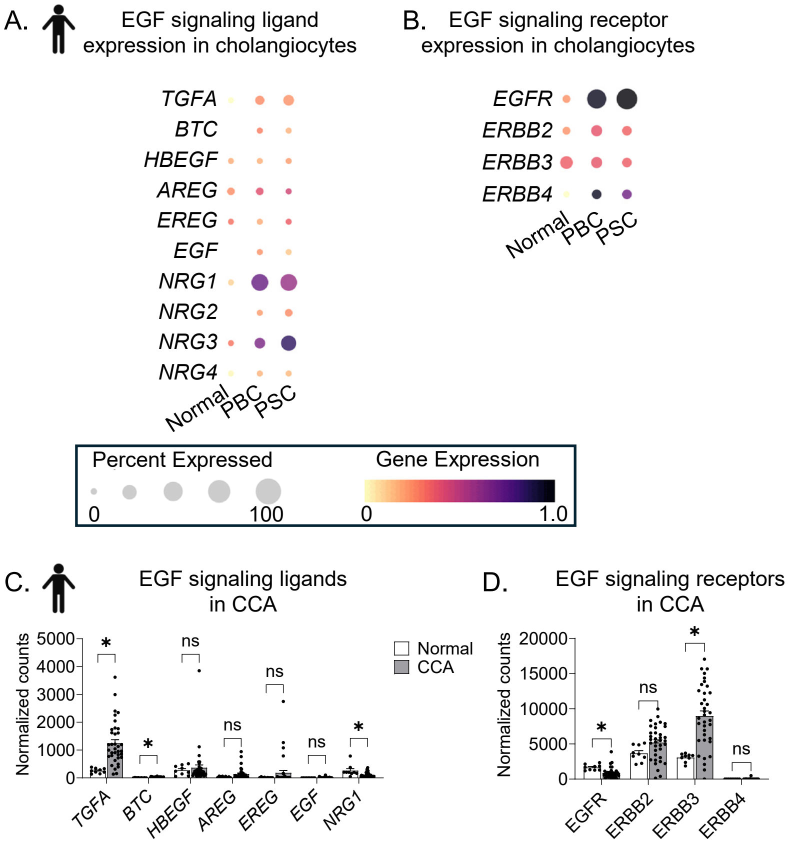

Suppl. Fig. 7: EGF signaling in human hepatobiliary tissues. Single cell analysis of EGF family ligands (A) and receptors (B) in intrahepatic cholangiocytes from primary biliary colitis (PBC) and primary sclerosing cholangitis (PSC) datasets using CellxGene software. Analysis of normal and cholangiocarcinoma (CCA) samples from The Cancer Genome Atlas for EGF signaling ligands (C) and receptors (D).

Antibody:	Host:	Company:	Catalog#:	Concentration:
**Primary:**				
KRT19	Rat	DSHB	Troma III	1:100
Phospho-EGFR (Tyr1068)	Rabbit	Invitrogen	44-788G	1:50
**Secondary:**				
Alexa Fluor 555	Goat	Invitrogen	A21428	1:1000
Alexa Fluor 555	Goat	Invitrogen	A21434	1:1000
Biotinylated	Donkey	Vector Laboratories	BA-9400	1:1000

Suppl. Table 1. Primary and secondary antibodies.

**Gene target:**	**Forward Primer:**	**Reverse Primer:**
18s	5’- GTAACCCGTTGAACCCCATT -3’	5'- CCATCCAATCGGTAGTAGCG -3'
Egfr	5'- GCCATCTGGGCCAAAGATACC -3'	5'- GTCTTCGCATGAATAGGCCAAT -3'
Erbb2	5'- CAGCTCGGAGACCTGCTATG -3'	5'- GTTCGTCCAGGTCCACACAT -3'
Erbb3	5'- TCGCCTGGATGTCCTCCTAA -3'	5'- GGTCACACTCAGCCCGTTTA -3'
Erbb4	5'- CAGATCAGGATCGGGAGTGC -3'	5'- TGGTAAAGTGGAATGGCCCG -3'
Tgfα	5’- GCACCCTGCGCTCGGAAGAT -3’	5’- TCTGGGATCTTCAGACCACT -3’
Btc	5'- AGCACAGTTGATGGACCCAA -3'	5'- CAGGAGGGAGTTTGCTCGTC -3'
Hbegf	5’- CGGGGAGTGCAGATACCTG -3’	5’- TTCTCCACTGGTAGAGTCAGC -3’
Ereg	5'- CAGCACAACCGTGATCCCAT -3'	5'- CAGACCAGTGTAGCCCACTT -3'
Areg	5’- GGGGACTACGACTACTCAGAG -3’	5’- TCTTGGGCTTAATCACCTGTTC -3’
Egf	5’- CCTGCCCCCTTCCTAGTTTTC -3’	5’- CTCCGTTCTGTTGGTCTACCC -3’
Nrg1	5'- TCTCATCCGAGGCATACACT -3'	5'- GTCCCAGTCGTGGATGTAGA -3'
Nrg2	5'- GCCAGATCCTAAGCAAAAGGC -3'	5'- GTGGTCTGTAGCTGGCACAT -3'
Nrg3	5'- CTATCAAGCACCACAGCCCA -3'	5'- AGCTGTATAGGCAGGTGGGA -3'
Nrg4	5’- GACTGTGGACCATACGACGA -3’	5’- GGCCAGTGATGACAGTAGCAG -3’

Suppl. Table 2. Quantitative reverse transcription polymerase chain reaction (qRT-PCR) primers.
